# Risk of manic switch with antidepressants use in patients with bipolar disorder in a Nigerian neuropsychiatric hospital

**DOI:** 10.4102/sajpsychiatry.v24i0.1215

**Published:** 2018-11-06

**Authors:** Ayodele L. Fela-Thomas, Osasu S. Olotu, Oluyomi Esan

**Affiliations:** 1 Department of Clinical Sciences, Federal Neuropsychiatric Hospital, Nigeria; 2 Department of Psychiatry, University of Ibadan, Nigeria

## Abstract

**Background:**

Depressive disorders are common among those with bipolar affective disorder (BAD) and may necessitate the use of antidepressants. This has been suggested to precipitate manic episodes in some patients.

**Objectives:**

This study aims to determine the prevalence of and factors associated with manic switch in patients with BAD being treated with antidepressants.

**Methods:**

Case notes of patients who were treated at a Nigerian neuropsychiatric hospital for a BAD from 2004 to 2015 were reviewed. BAD diagnosis was made using ICD-10 criteria. Treatment for bipolar depression included monotherapy (i.e. antidepressants, antipsychotics or mood stabilisers) or combination therapy (mood stabiliser with an antidepressant or a combination of mood stabilisers, antipsychotics and antidepressants). The primary outcome measure was a switch to mania or hypomania within 12 weeks of commencing an antidepressant.

**Results:**

Manic or hypomanic switch (MS) was observed in 109 (44.3%) of the participants. Female gender, younger age, number of previous episodes and a past history of psychiatric hospitalisation were all significantly associated with a risk of MS. There was no significant difference in the rate of MS in either those treated with adjunct antidepressants therapy with a mood stabiliser or an antipsychotic or those placed on a combination of antidepressants, antipsychotics and mood-stabilising agents.

**Conclusion:**

A large proportion of patients with BAD on antidepressants experience medication-induced manic or hypomanic switch.

## Introduction

Bipolar affective disorder (BAD) is a chronic debilitating illness with an estimated lifetime prevalence of 0.1% – 7.5%.^1^ The disorder is characterised by manic episodes or hypomanic episodes interspersed with depressive episodes.^2^ Bipolar depression (BD) is more pervasive; its treatment is more challenging than the manic phase and is associated with significant morbidity and mortality.^2,3,4^ A significant proportion of the burden of bipolar disorder is related to suicidal behaviours.^5,6^

Recent guidelines for the treatment of BD encourage the use of quetiapine, lithium or lamotrigine monotherapy or a combination of antidepressants with anti-manic agents (mood stabilisers or antipsychotics) when the depression is severe.^7,8,9^ However, the use of antidepressants in BD is controversial because it is believed to precipitate a manic switch, rapid cycling or mixed affective state.^10,11,12,13^ Some argue though that this risk is only associated with the monotherapy of antidepressants use.^14^ Findings from some studies suggest that the rate of manic or hypomanic switch (MS) in bipolar-depressed patients on adjunct antidepressants and mood stabiliser is not higher than that reported in those on a mood stabiliser monotherapy,^15,16,17^ whereas some argue that the addition of antidepressants in the treatment of BD confers no benefit.^10,18,19^ Existing reports suggest that the propensity of a manic switch by antidepressants is higher for the tricyclic antidepressants (TCAs) compared to the second-generation antidepressants (SGA),^12,20,21,22^ although this may have been confounded by sparse data on these classes of antidepressants compared to the TCAs.^23^ The question then is: can mania occur in BD when such patients are placed on adjuncts antidepressants? Is the rate of induction reduced when patients are placed on a combination of antidepressants and antipsychotics or antidepressants and mood stabilisers or when placed on all three classes of drugs? What factors are associated with this antidepressant-induced MS?

Although studies exist outside Africa on manic switch among patients with BD, in Africa, particularly Nigeria, there is a dearth of information on this subject.^19^ More so, the majority of these studies looked at manic switch in both unipolar and bipolar patients simultaneously with largely inconclusive results.^24^ We thus decided to focus on manic switch occurring solely among patients with BD.

This study therefore aims to estimate the prevalence of manic switch and determine the correlates of manic switch in patients with BD being treated with a combination of a mood stabiliser or antipsychotic and antidepressants in a neuropsychiatric hospital in Benin City, Nigeria.

As a secondary outcome, we also aimed to ascertain if the risk of manic switch was more likely in those placed on adjunct antidepressants therapy with either a mood stabiliser or an antipsychotic or those placed on a combination of antidepressants, antipsychotics and mood-stabilising agents.

## Methods

### Study population and design

Case notes of both adolescents and adults who received either inpatient or outpatient care for a BAD in Federal Neuropsychiatric Hospital, Benin City, during 2004–2015 were reviewed.

### Setting

The Federal Neuropsychiatric Hospital, Benin City, is a Federal Government-owned specialist hospital situated in South-Southern Nigeria. It is a 230-bed facility that provides specialist services regarding mental health to mentally ill children, adolescents and adults. Services offered include inpatient and outpatient care as well as emergency services to mentally ill persons across the region (a geographical catchment area of six states) of Nigeria. The hospital is located in Benin City, the capital of Edo state.

### Inclusion and exclusion criteria

Patients must have a primary diagnosis of BAD and currently under treatment or have received treatment at the hospital between 2004 and 2015. They must have experienced a depressive episode at some point during the treatment and must have been treated with an antidepressant during the depressive episode.

They must be on antidepressants for at least 3 months. This cut-off was used based on the suggestion that a risk of switch was more likely to occur at about 10 weeks from the initiation of antidepressants.^25^

Patients with co-morbid organic disorders and a schizoaffective disorder were excluded from the study.

### Sample size

Sample size was calculated using the following formula for population studies:^21,26^

- *N* = *Z*^2^*pq*/*d*^2^
- Where *Z* = the normal standard deviate 1.96 at 95% confidence interval.
- *p* = estimated prevalence of manic switch = 0.44.
- *q* = 1-*p* = 0.56.
- *d* = sampling error tolerated set at 0.05.
- Thus, the minimal sample size will be 194.

### Measures

Data on socio-demographic and clinical variables of the participants, such as age, age at time of switch, gender, marital status, level of education, number of manic or depressive episodes before current depressive episode, age at onset of first episode and duration of untreated psychosis (DUP – defined as the period between onset of first psychotic symptoms and initiation of first appropriate intervention for the psychosis; in this instance, commencement of antipsychotics),^27^ history of a previous psychiatric hospitalisation, patients’ current medication when the manic switch occurred (mood stabilisers, antipsychotics and antidepressants) were retrieved from the individual case notes.

A diagnosis of BAD was made based on International Classification of Diseases ICD-10 criteria (F31 code). Routinely, when clinical assessment is made at the emergency unit, diagnostic information is retrieved using the ICD-10 criteria. This is further confirmed by the attending consultant before a final diagnostic management is documented. At the point of data entry, this diagnosis was further crosschecked using the ICD-10 criteria by the research assistant who is a specialist registrar in psychiatry. The discharge summary diagnosis for the last admission was also compared with the diagnosis at admission to prevent misclassification. In cases where documented diagnosis was doubtful, it was clarified by A.L.F.T.

### Outcome

The outcome measure was an occurrence of a manic or hypomanic episode (using 1CD 10 criteria) within 12 weeks of commencing an antidepressant.^21^ Medication-induced MS was defined as an episode of mania, hypomania or mixed affective state (using the ICD-10 criteria [ICD code F30]) within the first 12 weeks of commencing antidepressants.^21^ We chose this cut-off to reduce the bias of being unable to delineate a switch from the natural course of the illness.

This definition included those who either switched directly from a depressive phase to a hypomanic or manic phase or fully remitted from depression but within the 12 weeks switched into a hypomanic or manic phase.

### Procedure

Data collection was performed by a specialist registrar in psychiatry who retrieved case notes of all patients diagnosed with BAD who either received inpatient or outpatient care in the hospital within 2004–2015. The diagnosis was crosschecked using the criteria for ICD-10 (F31 code). Those who met the inclusion criteria were subsequently separated for the assessment of an MS or mixed affective state (using the ICD 10 criteria; F30 code). In cases where outcome measure was not clear to the research assistant, this was reviewed with A.L.F.-T. who reconfirmed all diagnoses.

### Statistical analyses

Counts and percentages were employed to describe categorical variables, whereas continuous variables were described using means. To determine the association between categorical variables, chi-square test was carried out. Fisher’s exact test was employed where necessary. We analysed a subgroup consisting of (1) mood stabiliser + antidepressant and (2) antipsychotics + antidepressants combination while stratifying antidepressants into TCAs and selective serotonin reuptake inhibitors (SSRIs), to compare the rate of switch within the two classes. Level of significance was set at < 0.05.

## Ethical consideration

Ethical approval was obtained from the Research and Ethics Committee of the Federal Neuropsychiatric Hospital, Benin City.

## Results

### Profile of participants

A total of 862 case notes with a diagnosis of BAD were retrieved. Only 266 (30.9%) cases met the inclusion criteria. Twenty cases were discarded on account of incomplete data or doubtful diagnosis (occurrence of switch was doubtful or did not fulfil ICD-10 criteria for BAD or hypomania); 246 cases were thus included in the analysis.

The mean age was 35.9 (± 12.02) years and range was 14–74 years. The age at first presentation ranged from 11 to 65 years, mean 29.3 and s.d. ± 10.59. Females accounted for 69.9% of participants.

The majority of the participants (79.3%) were on or had been placed on a TCA. All participants were on antidepressants either as a monotherapy, in combination with either a mood stabiliser or an antipsychotic or a combination of mood stabiliser, antipsychotics and antidepressants. Eighty-six participants (35%) were on a combination of antidepressant, mood stabiliser and antipsychotic treatment. Thirty-four participants (13.8%) were on antidepressants and mood stabilisers, whereas 122 (49.6%) were placed on antidepressants and antipsychotics. Only 4 (1.6%) participants were on antidepressants alone. None were placed on either antipsychotics or mood stabiliser alone.

A switch from depression to either manic or hypomanic state was observed in 109 (44.3%) of the participants (Table 1).

**TABLE 1:** Socio-demographic and clinical variables.

Variable	*N*		%
**Gender *N* = 246**			
Male	74		30.1
Female	172		69.9
**Current age**			
11 to 20	15		6.1
21 to 30	88		35.8
31 to 40	67		27.2
41 to 50	48		19.5
➢ 50	28		11.4
Mean (s.d.)	35.90 (± 12.02)		
**Highest level of education *N* = 242**			
No formal education	19		7.7
Primary school	48		19.5
Secondary school	128		52.0
Tertiary	47		19.1
**Marital status *N* = 246**			
Married	107		43.5
Single	106		43.1
Separated or divorced or widow	33		13.4
**Age at first presentation *N* = 222**			
11 to 20	52		21.1
21 to 30	89		36.2
31 to 40	49		19.9
41 to 50	23		9.3
➢ 50	9		3.7
Mean (s.d.)	29.3 (± 10.59)		
**Previous episodes *N* = 221**			
1	13		5.3
2	67		27.2
3	85		34.6
➢ 3	56		22.8
**Age at time of switch *N* = 96**			
10 to 20	10	4.1	
21 to 30	31	12.6	
31 to 40	24	9.8	
41 to 50	19	7.7	
51 to 80	12	4.9	
Mean (± s.d.)	16.5 (± 20.40)		
**Past history of psychiatric hospitalisation *N* = 246**			
Yes	82	33.3	
No	164	66.7	
**Duration of untreated psychosis *N* = 239**			
None (0)†	129	52.4	
< 10 years	98	39.8	
10–20 years	2	0.8	
➢ 20 years	10	4.1	
Mean (s.d.) (months)	34.3 (± 73.58)		
**Antidepressant use *N* = 246**			
Amitriptyline	186	75.6	
Imipramine	9	3.7	
Citalopram	18	7.3	
Escitalopram	4	1.6	
Fluoxetine	10	4.1	
Paroxetine	3	1.2	
Sertraline	16	6.5	

† , None: Zero duration of untreated psychosis.

### Correlates of manic switch

Females were more likely to experience an MS (*p* = 0.001). Participants of 20 years of age or younger were significantly more likely to experience a switch (*p* = 0.035). Participants with a past history of psychiatric hospitalisation were significantly more likely to experience a switch (*p* < 0.001). Participants who had four or more prior episodes were more likely to experience a switch compared with those who had experienced fewer episodes (57.1%, *p* = 0.003).

We found no significant association between the marital status of the participants, age at onset of first episode, DUP and the type of antidepressant used with a risk of MS.

Although those on a combination of an antidepressant with mood stabilisers were numerically less likely to experience a switch (35.3%), we found no significant association between the three treatment combination groups and a risk of MS.

Among the antidepressant + mood stabiliser group, even though the rate of switch was numerically higher in the TCA group compared to the SSRI group, this was not of statistical significance (39.3% vs.16.7%). However, in the antipsychotics + antidepressant group, the rate of switch did not differ significantly between the two antidepressant groups (46.7%, respectively) (Table 2).

**TABLE 2:** Socio-demographic and clinical variables with the presence of switch.

Variable	Switch				*c*^2^	*p*
	Yes		No			
	*n*	%	*n*	%
**Gender**						
Female	88	51.2	84	48.8	10.89	0.001*
Male	21	28.4	53	71.6	-	-
**Marital status**						
Single	51	48.1	55	51.9	1.37	0.51
Married	43	40.2	64	59.8	-	-
Separated or divorced or widow	15	45.5	18	54.5	-	-
**Current age**						
11 to 20	12	80.0	3	20.0	10.30	0.035*
21 to 30	37	42.0	51	58.0	-	-
31 to 40	24	35.8	43	64.2	-	-
41 to 50	22	45.8	26	54.2	-	-
51 to 80	14	50.0	14	50.0	-	-
**Age at first presentation**						
11 to 20	31	59.6	21	40.4	9.34	0.05‡
21 to 30	31	34.8	58	65.2	-	-
31 to 40	19	38.8	30	61.2	-	-
41 to 50	11	47.8	12	52.2	-	-
51 to 80	5	55.6	4	44.4	-	-
**Past history of psychiatric hospitalisation**						
Yes	50	61.0	32	39.0	13.85	< 0.001*
No	59	36.0	105	64.0	-	-
None†	59	45.7	70	54.3	0.477	0.962‡
< 10 years	43	43.9	55	56.1	-	-
10 to 20 years	1	50.0	1	50.0	-	-
> 20 years	4	40.0	6	60.0	-	-
**Previous episodes**						
1	1	7.7	12	92.3	13.718	0.003*
2	27	40.3	40	59.7	-	-
3	47	55.3	38	44.7	-	-
> 3	32	57.1	24	42.9	-	-
**Type of antidepressant**						
TCA	89	-	106	54.4	0.676	0.254
SSRI	20	-	31	60.8	-	-
**Antidepressant alone**						
Yes	-	-	4	100.0	-	0.094‡
No	109	45.0	133	55.0	-	-
**Antidepressant, mood stabiliser and antipsychotics**						
Yes	40	46.5	46	53.5	0.260	0.687
No	69	43.1	91	56.9	-	-
**Antidepressant and mood stabiliser**						
Yes	12	35.3	22	64.7	1.299	0.170
No	97	45.8	115	54.2	-	-
**Antidepressant and mood stabiliser *N* = 34**						
TCA	11	39.3	17	60.7	-	0.389‡
SSRI	1	16.7	5	83.3	-	-
**Antidepressant and antipsychotics**						
Yes	57	46.7	65	53.3	0.571	0.265
No	52	41.9	72	58.1	-	-
**Antidepressant and antipsychotics *N* = 122**						
TCA	43	46.7	49	53.3	0.000	0.994
SSRI	14	46.7	16	53.3	-	-

SSRI, selective serotonin reuptake inhibitors; TCA, tricyclic antidepressant.

† , None: Zero duration of untreated psychosis;

‡ , FET.

* *p* < 0.05

## Discussion

A total of 44.3% of the participants with a BAD who were treated with antidepressants experienced an MS. Female gender, younger age, higher number of previous episodes of a bipolar disorder and a previous psychiatric hospitalisation were all significantly associated with the risk of an MS.

We found no significant difference in the rate of MS among those treated with a combination of antidepressants-mood stabiliser-antipsychotics or adjuncts antidepressants-mood stabiliser or antidepressants-antipsychotics combination.

The prevalence of MS reported in this study is in line with the result from a multicentre study in which a lifetime prevalence of 44.4% was reported.^21^ The prevalence of MS from our study is however higher than the 35% reported in two different studies.^17,22^ Methodological differences in these studies may account for this dissimilarity in results obtained. For instance, the study by Altshuller et al. was a randomised double-blind clinical trial conducted in two stages, a 10-week acute phase and a 1-year continuation phase. Moreover, it was a multicentre study which may have more diverse patient characteristics and consists of only adult samples as compared to this study comprising both adolescent and adult samples; thus, the results may not be comparable.^17^

We reported a higher risk of manic switch among the females and those aged 20 years or younger similar to what was reported in other studies.^10,13,22^ However, a prospective and controlled study of patients with bipolar disorder on antidepressants separately reported that sex and age did not influence the risk of experiencing antidepressant-induced mania in their participants.^28,29^ A plausible explanation may be the study design. A prospective study, for instance, controls for potential confounders within the study design as opposed to a retrospective one with methodological constraints. Other differences for this variation may also include the fact that Gao et al.’s study was a retrospective search of clinical trials data and was limited to antidepressant monotherapy in patients with a rapid cycling bipolar disorder and thus the results may not be comparable.^28^ The preponderance of manic switch in the female gender may reflect the prevalence of depression among this subgroup and the increased likelihood of being placed on an antidepressant therapy. More so, females are more likely to experience rapid cycling or a mixed state which are vulnerable factors in precipitating antidepressant-induced mania.^30,31,32^

A plausible explanation for the increased risk of manic switch in those below 20 years of age may be a reflection of a subgroup with specific vulnerability to manic induction; again, this may also be connected to the peak age at onset of bipolar disorder, which is reported to occur in adolescence.^33,34^ Early onset of BAD has been associated with a shorter duration of illness recurrence, fewer euthymic days and rapid cycling.^35,36^ These may all confer an increased vulnerability on such patients experiencing a manic switch with antidepressants usage.^10,30,35^

We found a higher risk of manic switch in participants with multiple previous episodes compared to single episodes. This finding is at variance with the results obtained in a prospective controlled study of antidepressants use among patients with BD.^11^ In this study, structured diagnostic interviews were used to reconfirm initial diagnosis of a bipolar disorder, a procedure that was not done in this study, although all retrospective diagnoses were made based on ICD-10 criteria. Moreover, the age categories of participants in their study (18–65 years) differ from the participants in this study.^11^ Our result, however, is in tandem with the results obtained in a study comparing bipolar patients with treatment emergent affective switch and those experiencing spontaneous switch. A plausible explanation is that those with multiple previous episodes were more likely to experience a rapid cycling course,^23^ thus conferring on them an increased susceptibility of experiencing a manic switch. Evidence exists from several studies of the increased switch rate observed in rapid cycling bipolar-depressed patients treated with or without antidepressants.^10,37,38^

A past history of psychiatric hospitalisation was significantly associated with a risk of manic switch. This perhaps may suggest severity of such past episodes, a factor that has been reported as significantly associated with manic switch in patients with bipolar disorder.^10,39^ Again, those that experienced a switch may more likely be hospitalised.

Contrary to the findings in a number of studies, we found no significant association between the type of antidepressants used and the risk of experiencing a manic switch.^22,39^ Our inability to detect a significant difference may be connected to differences in methodology of the studies. For instance, in the study by Mundo et al., diagnostic subtypes of BAD were considered. However, the small sample size reported as a limitation of their study may have influenced their findings.

We found no significant difference in the rate of MS between the antidepressants-mood stabiliser, antidepressant-antipsychotics, or antidepressant-antipsychotics and mood stabiliser combination groups, even though we observed that the rate of MS in the mood stabiliser-antidepressant group was less compared to the other two groups. Two retrospective naturalistic studies separately reported that mood stabilisers as adjunct to antidepressants in BD significantly reduced the risk of antidepressant-induced transition,^14,40^ although a meta-analysis by Tondo et al. on mania associated with antidepressant treatment did not find a clear-cut benefit of adjunct mood stabiliser with antidepressant treatment in the prevention of antidepressant-induced MS.^12^ The absence of lithium among the mood stabilisers considered in our study may be a plausible explanation for our inability to detect a significant difference between the groups.

## Limitations

There were no objective means of differentiating medication-induced MS from the natural transition to mania in BAD. Again, the study does not consider poor adherence by some participants, a factor that may confound the results of our study. The lack of controls, non-randomisation and absence of blinding are all significant limitations of this study. Being a single-site study, it also limits the generalisability of our findings. Again, causality cannot be inferred, being a retrospective study.

## Conclusion

We reported a prevalence of 44.3% MS among a cohort with BD being treated with antidepressants.

We also observed that female gender, younger age, an increased number of previous episodes and a prior history of hospitalisation were all associated with the risk of experiencing a medication-induced manic switch. It is recommended that caution be taken in administering antidepressants to those patients perceived to be at risk of such transition. The retrospective nature of the study clearly limits our findings as it only suggests an association. However, a prospective controlled study is recommended for future research on this subject to affirm our results and further shed light on the role of antidepressants combination type with risk of manic switch in bipolar-depressed patients.
